# Advances in 3D Inner Ear Reconstruction Software for Cochlear Implants: A Comprehensive Review

**DOI:** 10.3390/mps7030046

**Published:** 2024-05-23

**Authors:** Michail Athanasopoulos, Pinelopi Samara, Ioannis Athanasopoulos

**Affiliations:** 1 Otolaryngology-Head & Neck Surgery, Athens Pediatric Center, 15125 Athens, Greece; miathanasopoulos@gmail.com (M.A.); dr.iathanasopoulos@gmail.com (I.A.); 2 Children’s Oncology Unit “Marianna V. Vardinoyannis-ELPIDA”, Aghia Sophia Children’s Hospital, 11527 Athens, Greece

**Keywords:** cochlear implants, hearing loss, 3D inner ear reconstruction software, segmentation, OTOPLAN

## Abstract

Auditory impairment stands as a pervasive global issue, exerting significant effects on individuals’ daily functioning and interpersonal engagements. Cochlear implants (CIs) have risen as a cutting-edge solution for severe to profound hearing loss, directly stimulating the auditory nerve with electrical signals. The success of CI procedures hinges on precise pre-operative planning and post-operative evaluation, highlighting the significance of advanced three-dimensional (3D) inner ear reconstruction software. Accurate pre-operative imaging is vital for identifying anatomical landmarks and assessing cochlear deformities. Tools like 3D Slicer, Amira and OTOPLAN provide detailed depictions of cochlear anatomy, aiding surgeons in simulating implantation scenarios and refining surgical approaches. Post-operative scans play a crucial role in detecting complications and ensuring CI longevity. Despite technological advancements, challenges such as standardization and optimization persist. This review explores the role of 3D inner ear reconstruction software in patient selection, surgical planning, and post-operative assessment, tracing its evolution and emphasizing features like image segmentation and virtual simulation. It addresses software limitations and proposes solutions, advocating for their integration into clinical practice. Ultimately, this review underscores the impact of 3D inner ear reconstruction software on cochlear implantation, connecting innovation with precision medicine.

## 1. Introduction

Hearing impairment poses a significant challenge for individuals on a global scale, impacting their daily lives and social interactions. Cochlear implants (CIs) have emerged as a revolutionary solution for severe to profound hearing loss, capturing and processing external sounds to deliver electrical signals directly to the auditory nerve through an implanted electrode array [1]. Next to patient-specific factors such as age, etiology and duration of deafness, and cognitive capabilities, the outcome of CI procedures relies significantly on minimally traumatic surgical techniques and correct electrode choice to match the individual anatomy [2,3,4]. Therefore, an accurate pre-operative planning and post-operative evaluation of electrode position is indispensable. This emphasizes the role of advanced three-dimensional (3D) inner ear reconstruction software in this complex process. Pre-operative imaging stands as a key step in patient selection for CI surgery, necessitating a thorough investigation of imaging findings that can influence the surgical approach. Precisely identifying significant anatomical landmarks such as the skull base level, facial nerve, sigmoid sinus, chorda tympani, fossa incudis, and the opening of the round window during CI operations holds considerable importance, as it substantially contributes to the overall success and precision of the procedure [5]. In particularly delicate zones, surgeons navigate within a slim margin of a few millimeters, underlying the importance of preserving this limited safe working distance to prevent potential surgical trauma. This pre-surgical assessment has diverse objectives, encompassing the evaluation of cochlear deformities, ensuring the integrity of intra-cochlear structures to retain residual hearing, and the meticulous selection of an appropriate electrode. Disparities in cochlear volume [6], combined with individual anatomical variations in the normal cochlea, directly influence its size and length. Histologic studies report discrepancies in the length of the organ of Corti that range from 25 to 35 mm [7]. Consequently, selecting an optimal electrode requires the use of a transparent and reliable tool, for assisting the surgeon in scrutinizing the inner ear’s morphology and precisely measuring cochlear dimensions.

In a more in-depth analysis, advanced tools like 3D Slicer, Amira, Mimics, and OTOPLAN are pivotal in the pre-operative planning stage, providing otosurgeons with detailed representations of cochlear anatomy [8]. Beyond visualizing intricate cochlear structures, these programs offer robust support for virtual simulations [9]. This dynamic functionality enables surgeons to explore various implantation scenarios, comprehensively assessing potential challenges, refining surgical strategies, and optimizing approaches well in advance of the actual procedure. Utilizing these tools enhances surgical readiness by promoting a thorough understanding of complex details, resulting in a reduction in procedural risks.

Post-operative scans are equally crucial, as they play an essential role in identifying complications like electrode array dislocation, folding, and malposition, as well as ossification, fibrosis, and granulation tissue formation [10]. These scans empower medical professionals to effectively manage potential post-surgical complications, ensuring the sustained effectiveness and longevity of CIs. High-resolution images allow surgeons to promptly address abnormalities that may compromise CI function. In case of migration or malposition, corrective surgical interventions can be planned to reposition the electrode array inside the cochlea. For electrode malfunction, replacement of the malfunctioning electrode or the entire CI system may be required. Infections can be treated with appropriate antibiotic therapy, and ossification, fibrosis, or granulation tissue formation may necessitate surgical intervention to remove the tissue and restore proper function. This proactive approach facilitates timely intervention to mitigate complications. Moreover, post-operative CT scans enable ongoing monitoring of the healing process and identification of any delayed complications, enabling prompt intervention and management as needed.

Advancements in software, featuring intricate image segmentation techniques and advanced 3D modeling, have the potential to elevate CI techniques [11]. These innovations not only boost procedural accuracy but also seamlessly integrate with established imaging modalities such as computed tomography (CT) and magnetic resonance imaging (MRI) [12]. This combination of technology and medical science represents a noteworthy improvement in tackling hearing impairment, introducing a new level of complexity and precision to the field.

Despite technological progress, challenges persist in this domain. This review transcends technical exposition, delving into the evolution of 3D inner ear reconstruction software tailored for CIs. It traces the historical development of these tools, emphasizing features like image segmentation, 3D modeling, and virtual simulation capabilities. Through relevant studies, the aim is to underscore the benefits and outcomes arising from the use of 3D inner ear reconstruction in CI procedures. This review critically evaluates current software limitations and challenges, proposing potential solutions. Regulatory considerations and the necessity for standardization in software development are examined, providing a practical perspective on their integration into clinical practice. Beyond technological aspects, the review evaluates the applied clinical applications of these tools in surgical planning for CIs. Their incorporation into clinical practice represents a significant stride forward, embodying the convergence of innovation, compassionate care, and precision medicine in the realm of cochlear implantation.

## 2. Historical Development of 3D Inner Ear Reconstruction Software: Pioneering Milestones, Breakthroughs, and Technological Progress

The historical trajectory from 2D to 3D imaging in the context of ear anatomy has been a revolutionary narrative, reshaping the landscape of otolaryngology and ear reconstruction. This transformation introduces unprecedented levels of precision and comprehension in the diagnosis and treatment of ear-related conditions. In the initial stages of medical imaging, 2D modalities, such as traditional X-rays, were useful for detecting abnormalities or conditions affecting the bony structures of the ear, such as fractures, tumors, or anomalies in bone density. However, the intricate and 3D nature of the ear’s anatomy posed challenges in achieving comprehensive visualization [13]. The emergence of CT and MRI marked a pivotal advancement, offering cross-sectional views that allowed clinicians to delve deeper into the complexities of the ear [14]. Despite this evolution, reconstructing 3D structures from sectional images remained a skillful task for healthcare professionals.

The arrival of true 3D imaging technologies, coupled with sophisticated software, ushered in a new era for ear-specific diagnostics and surgical planning. Image segmentation techniques became instrumental, enabling precise identification and isolation of crucial ear structures with unparalleled detail. This development proved impactful in the field of ear reconstruction, where a thorough understanding of intricate anatomy is paramount. For surgeons specializing in otological procedures, the integration of 3D modeling became a groundbreaking tool. This innovation facilitated the creation of patient-specific 3D representations of the ear, allowing for a thorough exploration of spatial relationships and anatomical nuances. Surgeons could now engage in more precise pre-operative planning for interventions such as cochlear implantation or reconstructive ear surgery [15].

The addition of virtual simulation capabilities further elevated this development, revolutionizing the training and preparation of otolaryngologists. Virtual environments enabled practitioners to simulate intricate ear surgeries, refining their skills and techniques in a risk-free digital space [16]. The integration of 3D imaging, image segmentation, and virtual simulation not only enhanced surgical precision, but also contributed significantly to the ongoing advancement of ear-specific medical practices.

## 3. Technological Features of Leading 3D Inner Ear Reconstruction Software: Pros and Cons

The integration of 3D software in the realm of ear reconstruction offers numerous benefits alongside a set of nuanced challenges.

On a positive note, the precision and customization capabilities of 3D technology empower surgeons to plan procedures, tailoring each reconstruction to the distinct anatomical features of individual patients. The enhanced visualization afforded by 3D models facilitates a deeper comprehension of intricate anatomical structures, enabling surgeons to navigate spatial complexities with greater confidence and accuracy during pre-operative planning. Sugarova et al. have recently introduced a robust method for accurately capturing the true cochlear basal turn, with significant clinical implications, utilizing a dataset of 1932 head scans. By employing 3D segmented inner ear images, they ensured precision across anatomical variations. The identification of ossification in the scala tympani aids in surgical planning, and their discovery of variation in the cochlear basal turn shape provides insights into electrode insertion ease [8]. Furthermore, the ability to communicate procedural details to patients is improved, fostering a more comprehensive understanding of the anticipated outcomes. Beyond the operating room, 3D models serve as tools for educating and training medical professionals, fostering a dynamic learning environment. The potential for reduced operating times and optimized resource utilization adds an efficiency dimension to the advantages.

However, challenges include the considerable cost associated with acquiring and implementing advanced 3D technology [17], as well as potential accessibility limitations in certain healthcare settings. Learning to navigate sophisticated software requires dedicated training, and integration into existing healthcare systems may present obstacles. Concerns regarding data security and privacy underscore the need for robust safeguards in handling patient information within the 3D modeling context. Balancing the utilization of technology with the preservation of the surgeon’s clinical judgment and experience is crucial to ensure optimal outcomes. Technical limitations, such as resolution issues, are already acknowledged and addressed in the ongoing development and adoption of 3D software for ear reconstruction.

## 4. Diverse Software Tools: Expanding Operational Capabilities in Inner Ear Reconstruction

There are several 3D reconstruction software tools used for imaging and modeling the ear. Some commonly used software includes Amira, 3D Slicer, Mimics, Simpleware ScanIP, OsiriX, ITK-SNAP, Blender, Visualization Toolkit (VTK), and Seg3D.

Amira (Thermo Fisher Scientific, Waltham, MA, USA) is a 3D visualization and analysis software used in life sciences and biomedical research [18]. It supports the reconstruction and visualization of complex anatomical structures, including the cochlea and inner ear, based on imaging data. 3D Slicer is an open-source software platform (BSD-style license) for medical image informatics, image processing, and 3D visualization. It is used by researchers and clinicians to analyze and visualize medical images including the ear [19]. Mimics (Materialise, Leuven, Belgium) is a versatile medical imaging software that allows for the segmentation and 3D reconstruction of anatomical structures from medical imaging data. It is widely used in the field of medical image processing and can be applied to reconstruct the cochlea and inner ear [20]. Simpleware ScanIP (Synopsys, Sunnyvale, CA, USA) is a software package that facilitates image-based modeling and simulation. It is used in biomedical research for converting medical imaging data into high-quality 3D models [21]. OsiriX is an open-source Digital Imaging and Communications in Medicine (DICOM) viewer for the Apple MacOS operating system with advanced post-processing capabilities [22]. While primarily known for its diagnostic imaging capabilities, it can also be used for 3D reconstruction of anatomical structures, including the cochlea and inner ear.

ITK-SNAP is a free, open-source software application for medical image segmentation, distributed under the GNU General Public License [23]. It provides tools for manual and semi-automatic segmentation and has been used in the reconstruction of various anatomical structures, including the inner ear. Blender is a free and open-source 3D computer graphics software, initially developed for animated films, visual effects, and video games. It is versatile and widely used in various fields, including biomedical visualization. Blender allows for the creation of detailed 3D models and animations [24]. VTK (Kitware, Clifton Park, NY, USA) is an open-source software system for 3D computer graphics, image processing, and visualization [25]. It provides a platform for researchers and developers to create customized tools for visualization, making it applicable to cochlea and inner ear reconstruction. Seg3D is an open-source volume segmentation and processing tool designed for medical imaging applications, developed by the NIH Center for Integrative Biomedical Computing at the University of Utah Scientific Computing and Imaging (SCI) Institute. It provides advanced segmentation algorithms and 3D visualization tools, making it useful for the reconstruction of anatomical structures [26].

It should be noted that none of the software discussed above is classified as a medical device. Generally, researchers and clinicians may select different software tools based on their specific needs, preferences, and the nature of the imaging data they are analyzing [27]. It is also common for multiple software tools to be utilized in conjunction for different steps of the reconstruction process.

**Figure 1:**
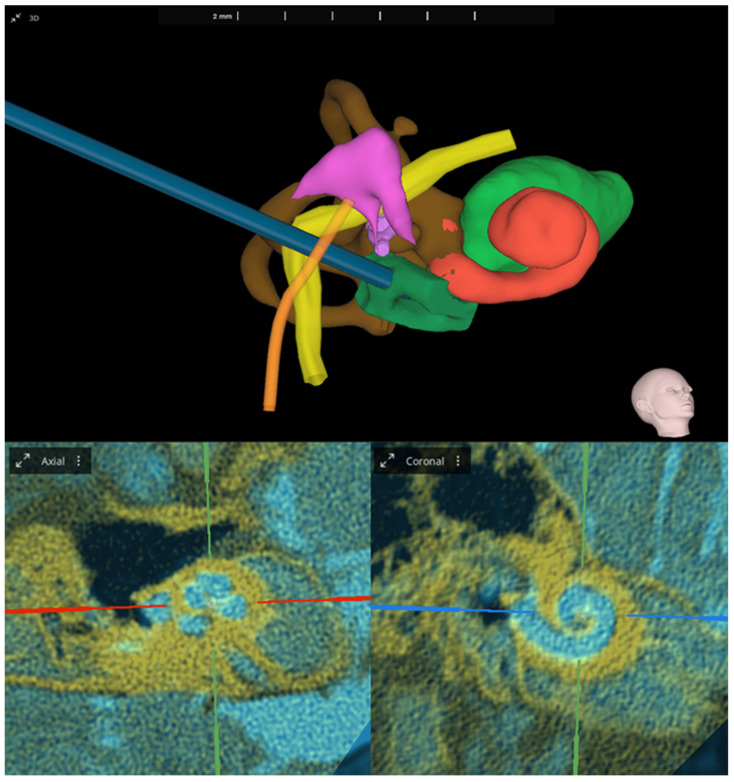
(**Top**): Three-dimensional inner ear reconstruction with optimal trajectory calculation. Shown anatomical structures: cochlea (red), inner auditory meatus (light green), vestibulum and semicircular canals (brown), facial nerve (yellow), chorda tympani (orange), ossicle chain (purple), and bony overhang (dark green). (**Bottom**): Image fusion showing 0.60 mm CT (yellow) and T2 weighted MRI (blue) in the oblique coronal view. Analyses and images were generated with OTOPLAN V4 (3.0.0).

Among the innovative systems in otological planning is OTOPLAN, a software developed jointly by CASCINATION AG (Bern, Switzerland) and MED-EL (Innsbruck, Austria) [28]. The software, introduced in 2018, represents an advancement in the pre-surgical planning process for MED-EL CIs. This exclusive software constructs detailed 3D models of inner ear structures, including the cochlea, round window membrane, bony overhang, semicircular canals, and internal auditory canal, assisting surgeons in visualizing the precise placement of each electrode array (Figure 1). Users can also zoom in on the 3D reconstruction and rotate it in any direction, offering a complete 360° view. By leveraging a robust 3D modeling algorithm, OTOPLAN ensures accurate visualization, enabling surgeons to assess delicate cochlear structures before intervention and strategize optimal outcomes, tailored to individual anatomical conditions. OTOPLAN provides extensive capabilities for integrating various types of pre- and post-operative images (CT, MRI). These include fusing CT and MRI scans to visualize all relevant tissue types—bones, nerves, and soft tissue—or merging post-operative CT images with pre-operative cochlear measurements. This eliminates the need for audiologists to re-measure after implantation, streamlining the process and enhancing efficiency. Furthermore, image fusion is a tool for assessment of pathological findings, like vestibular schwannoma or intra-cochlear fibrosis, hence exceeding the software capabilities to be used strictly for cochlear implantation.

Seamlessly integrated into the HEARO system for robotic cochlear interventions [29], OTOPLAN aligns insertion angles with natural cochlear anatomy, facilitating smooth electrode array insertion into the scala tympani, while preserving critical structures such as the facial nerve and chorda tympani. Additionally, it provides an electrode visualization tool, enabling surgeons to assess compatibility with the patient’s unique cochlear structure and evaluate insertion depth and tonotopic pitch match for each electrode array [30]. Beyond the operating room, OTOPLAN enhances patient engagement by simplifying discussions about ideal electrode choices and individual surgical considerations. Its one-step data export feature automatically generates comprehensive case reports in PowerPoint format, aiding thorough consultations before and after intervention for a seamless and informed patient experience. The cochlear duct length (CDL) ranges from 30.7 to 43.2 mm, across studies [31], emphasizing the significance of electrode length in pitch-place matching. Optimal outcomes necessitate electrode coverage of at least two turns of the cochlea; failure to achieve this compromises pitch-place matching accuracy, resulting in suboptimal auditory perception characterized by robotic, tinny, or mechanical sound qualities. Thus, ensuring proper electrode coverage is imperative for enhancing the overall quality of the patient’s auditory experience. Finally, OTOPLAN automatically identifies electrode contacts, implant housing, and electrode leads on the CT scan, allowing for post-operative analysis and quality checking of lead management. This information can also assist in placing the second implant as symmetrically as possible on the opposite side. Another important aspect to consider is the value of anatomy-based fitting (ABF), which can be derived from post-operative CT analyses. OTOPLAN utilizes the position of each electrode contact to reallocate the frequency band filters of the audio processor in such a way to match the tonotopicity of the cochlea [32]. This approach tailors the device’s settings to individual anatomical variations, leading to improved speech recognition scores and patient sound acceptance [33,34].

So far, OTOPLAN may be the only medical device software on the market; however, there are other research software tools that need to be mentioned at this point. In 2022, Oticon Medical presented Nautilus—a web-based research platform for automatic pre-operative and post-operative cochlear analysis [35]. By implementing deep learning and Bayesian inference approaches, Nautilus characterizes cochlear structures from pre-operative clinical CT scans. By combining pre- and post-operative images, a set of personalized metrics for the exploration of clinically relevant aspects of CI therapy can be derived. For instance, electrode insertion trajectory can be modeled to aim for a specific insertion coverage. Post-operatively, Nautilus enables electrode array detection and anatomo-physiologically tuned fitting.

In 2023, Geiger and colleagues [36] introduced a novel imaging software developed by Advanced Bionics for analyzing pre- and post-operative cone beam CT (CBCT) scans. The software reportedly provides fast and accurate assessment of electrode scalar location within the cochlea, categorizing electrode insertion as scala tympani insertion, interaction with the basilar membrane, or scalar translocation with very high specificity. Comparison of semi-automatic and manual measurements for translocation detection shows similarity to interobserver variability, making the tool suitable for clinical implementation [37].

## 5. Integration of 3D Inner Ear Reconstruction Software with Various Imaging Modalities

The evolution of CI surgery has been profoundly shaped by the integration of advanced imaging technologies, notably CT and MRI. Various types of CT and MRI scans are used for 3D reconstruction in CI surgery. These include high-resolution CT (HRCT), CBCT, T2-weighted MRI, and T1-weighted MRI. These modalities, distinguished by their specific strengths, contribute to the systematic assessment and strategic planning for individuals contemplating CIs. Notably, micro-CT is gaining traction in clinical studies involving human temporal bone specimens, showcasing its potential for enhancing our understanding of cochlear anatomy. Nevertheless, the integration of micro-CT imaging into clinical practice remains unclear. Given its high dose, micro-CT imaging does not seem suitable for patient use; instead, it is primarily employed for training and validating automatic segmentation algorithms [38].

CT excels in capturing detailed information about bony labyrinth anomalies, particularly in conditions such as enlarged vestibular aqueduct (EVA). Its advantages include not typically requiring sedation and applicability to adults and post-lingually deafened individuals [39]. On the other hand, MRI is instrumental in soft tissue visualization, making it a valuable tool for identifying issues like labyrinth ossification and assessing cochlear nerve caliber. This capability is especially crucial for early detection of labyrinth ossification, notably in cases related to meningitis. MRI can also reveal aplastic or hypoplastic nerves even in the presence of a structurally normal cochlea [5]. MRI’s notable strength lies in its capacity to visualize the fluid content of the membranous labyrinth. Conditions such as a history of meningitis, temporal bone fracture, or otosclerosis may result in cochlear fibrosis or scarring, presenting as a discernible loss of fluid signal [14]. However, the use of MRI in pediatric patients may pose challenges due to the need for sedation. While CT can identify cochlear sclerosis, MRI outperforms in capturing the early fibrotic stages that may elude detection with CT alone [40]. Furthermore, the introduction of contrast enhancement in MRI serves as a valuable adjunct for supporting the diagnosis of fibrosis and inflammation. Both imaging modalities are suggested for all pediatric candidates [39].

**Figure 2:**
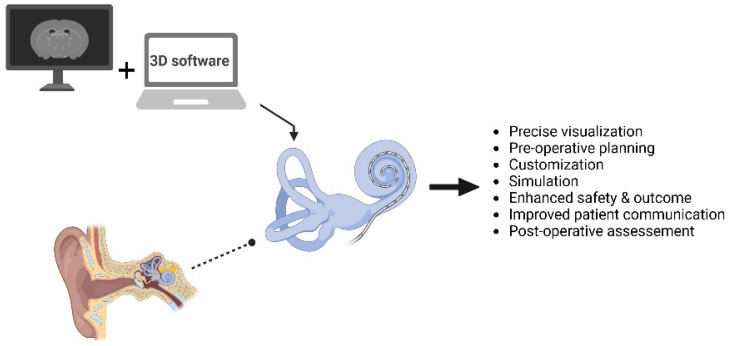
Schematic representation of the integration of established imaging modalities (like CT and MRI) with advanced 3D inner ear reconstruction software in cochlear implantation [created with https://app.biorender.com/illustrations (accessed on 5 April 2024)].

The integration of these two imaging modalities in clinical practice offers a comprehensive understanding of both soft tissue and bony structures relevant to CI surgery. Moreover, combining CT and MRI data with advanced 3D software provides additional insights beyond what traditional imaging alone can reveal (Figure 2). This enhanced visualization is particularly beneficial when assessing the intricate details of the cochlear anatomy, such as the cochlear nerve, labyrinth, and surrounding soft tissues. Moreover, 3D reconstruction facilitates a more detailed examination of the bony labyrinth anomalies detected by CT. It allows surgeons to rotate and manipulate the reconstructed model, gaining a holistic understanding of the spatial configuration and potential variations in the anatomy. This capability proves invaluable in identifying subtle abnormalities that may impact the surgical approach or patient outcomes.

Additionally, the integration of 3D reconstruction with CT and MRI data aids in simulating surgical scenarios. Surgeons can virtually navigate through the reconstructed model, practicing and refining their approach before the actual procedure. This virtual rehearsal contributes to improved surgical precision, reduces potential complications, and enhances overall procedural efficiency. Indeed, Ciodaro and colleagues [41] demonstrated the superiority of 3D volume rendering over traditional HRCT in post-operative evaluation of CI placement. Three patients underwent HRCT with and without 3D volume rendering, revealing improved identification of array malposition and complete migration out of the cochlea with rendering reconstruction. The study concludes that 3D rendering provides more detailed diagnostic information than HRCT, offering clearer representation of the cochlear topography and facilitating better post-operative assessment of implant placement.

The impact of high-resolution imaging on reconstructed models is far-reaching, as it minimizes distortions and artifacts inherent in the imaging process. This reduction ensures that reconstructed models faithfully mirror actual anatomical structures, contributing to precision in both diagnostics and planning. Moreover, high-resolution imaging enables the creation of patient-specific models, capturing the unique anatomical characteristics of individuals. It not only enhances the detailed representation of anatomical structures but also augments the practicality of these models across diverse applications, spanning medical diagnostics to surgical planning.

## 6. Clinical Applications and Surgical Planning Utilizing 3D Inner Ear Reconstruction Software, with a Focus on OTOPLAN

Recent studies conducted within the last five years have showcased impressive results, demonstrating the effective implementation of OTOPLAN within patient populations and highlighting its benefits in clinical contexts. These studies are nicely presented in Gatto’s review [42].

Lovato and de Filippis performed successful cochlear implantation on a patient with post-meningitis profound hearing loss and suspected cochlear ossification, eleven years after symptom onset. Despite conventional CT imaging initially deeming CI unsuitable, detailed examination with OTOPLAN revealed a viable approach. Surprisingly, no ossification was found around the round window/basal turn. They implanted a standard 19 mm contour array, achieving complete insertion. They advocated for OTOPLAN’s integration into routine CI evaluations, as it aids in visualizing cochlear turns and facial nerve pathways, potentially influencing electrode selection for optimal insertion depth and frequency stimulation [43].

In a study led by Yoshimura et al. [44], 105 Japanese patients with normal cochlea were analyzed to assess the importance of CDL in CI surgery. Utilizing OTOPLAN software, CDL measurements were taken pre-operatively to estimate the angular insertion depth (AID) of CI electrodes. Post-operative radiographic evaluations demonstrated a strong correlation between estimated and actual AID. These findings emphasize the critical role of accurate CDL assessment in optimizing CI electrode array selection for improved post-operative speech perception outcomes.

Garrada and colleagues explored the relationship between skin flap thickness, patient age, and the magnet strength used in the inductive link coil of the external processor among 57 CI patients, with an average age of 7.98 years. Employing OTOPLAN, their results imply that the selection of magnet strength could be influenced by both patient age and skin flap thickness [45].

A study conducted by Távora-Vieira and colleagues [46] explored the variances in auditory outcomes among patients who received CIs with arrays recommended by the OTOPLAN software versus those with alternative array selections. The study cohort consisted of 114 patients, with their pre-operative CT scans examined, to compare array recommendations with the actual implants in terms of type and length. The results highlighted that the majority of patients (83%) received more flexible arrays for reasons pertaining to structural integrity and hearing preservation. Conversely, patients with specific cochlear conditions necessitated stiffer arrays. Despite a subtle inclination among clinicians towards shorter arrays, the study suggests that integrating 3D imaging into pre-operative planning could bolster confidence in selecting longer arrays when deemed essential for achieving optimal hearing outcomes.

Another study conducted in pediatric patients highlighted the significant variability in cochlear size among Chinese CI candidates, underscoring the necessity for diverse electrode array lengths. The study showcased the capability of OTOPLAN software to detect malformations like incomplete partition type II, which may not be readily identified using conventional CT scans, thereby advocating for tailored electrode array selection [47].

The efficacy of 3D segmentation software for quantifying vestibular aqueduct (VAD) and inner ear volumes was also examined, alongside their correlation with linear VAD measurements. The analysis involved data from 21 children diagnosed with Mondini dysplasia (MD) and EVA who underwent cochlear implantation. The results revealed noteworthy associations between various factors and CT VAD, as well as inner ear volumes. Gender and VAD width at the midpoint were identified as significant predictors of gusher risk, emphasizing the crucial role of accurate measurement techniques in evaluating cochlear anomalies and anticipating surgical outcomes in pediatric MD and EVA cases [48].

Hajr and colleagues [49] sought to enhance electrode trajectory in CI surgery using OTOPLAN’s 3D models and assess the surgical distance of the retro-facial approach to the round window. Evaluation of CT scans from 25 cases indicated that the retro-facial approach was optimal for 52% of cases, with advantageous safety distances from the facial nerve. OTOPLAN imaging enabled meticulous planning and comparison with the standard facial recess approach, potentially improving future CI surgeries and extending the use of the retro-facial approach in other ear surgeries.

OTOPLAN software was also utilized in cases of advanced otosclerosis to facilitate surgical planning [50]. In a 73-year-old man with bilateral far-advanced otosclerosis, pre-operative radiologic assessments, including temporal bone CT and MRI alongside OTOPLAN, confirmed severe cochlear alterations. The software provided detailed insights into cochlear anomalies. Subsequent unilateral cochlear implant surgery with a perimodiolar implant on the left ear yielded positive outcomes without complications. The case suggests OTOPLAN’s utility in intricate otosclerosis cases, aiding in precise surgical planning and potentially improving surgical outcomes [51].

## 7. Challenges and Limitations in 3D Inner Ear Reconstruction Software: Solutions, Advancements, and Future Directions

Existing 3D inner ear reconstruction software faces significant challenges and limitations, primarily due to the complexity of the inner ear structure and the variability among individuals. One major challenge is the accurate segmentation of intricate anatomical features, such as the cochlea and vestibular system, from medical imaging data with varying resolutions and qualities. Additionally, the lack of standardized protocols for data acquisition and processing hinders interoperability and reproducibility. To address these challenges, ongoing research focuses on developing robust algorithms for automatic segmentation and registration, leveraging machine learning techniques to enhance accuracy and efficiency [52].

Advancements in computational resources and imaging technologies offer promising opportunities for real-time visualization and personalized modeling, facilitating pre-operative planning and surgical simulation [53]. The adoption of cloud-based platforms and artificial intelligence-driven workflows holds promise for streamlining data management and analysis, enabling seamless integration of patient-specific information into clinical decision-making processes. Furthermore, interdisciplinary collaborations between engineers, clinicians, and biomedical researchers are crucial for translating technological innovations into clinical practice, paving the way for improved diagnosis and treatment of inner ear disorders. Future directions entail refining existing methodologies, integrating multimodal imaging modalities, and exploring virtual reality-based platforms for immersive training and patient education, ultimately advancing the field of otological surgery and patient care.

In addition to the challenges mentioned, another significant hurdle in existing 3D inner ear reconstruction software is the accurate representation of tissue interfaces and microstructures within the inner ear. These fine details are crucial for understanding the pathology of various inner ear disorders and optimizing surgical interventions. However, current software often struggles with capturing these intricacies due to limitations in imaging resolution and processing algorithms. To overcome this, researchers are exploring novel imaging techniques such as high-resolution micro-CT [54] and MRI with advanced contrast agents [55] to enhance the visualization of inner ear structures at the cellular level. Furthermore, advancements in computational modeling, including finite element analysis and fluid dynamics simulations, enable more realistic biomechanical simulations of the inner ear’s dynamic behavior under pathological conditions or during surgical manipulations.

## 8. Regulatory Considerations and Standardization

Supervisory authorities such as the European Union’s Medical Device Regulation (MDR) and the U.S. Food and Drug Administration (FDA) play a crucial role in ensuring the safety and effectiveness of medical devices, including software applications like 3D inner ear reconstruction software [56,57]. These entities establish specific pathways for approval, which include rigorous evaluation processes to assess the product’s safety, effectiveness, and quality. Compliance with MDR certification or FDA clearance signifies that a device has met the necessary regulatory standards. Additionally, adherence to international quality management standards ensures that manufacturers have implemented robust quality management systems throughout the device’s lifecycle. Furthermore, ongoing monitoring and assessment post-market-approval are essential to ensure continued safety and effectiveness. Collaboration among developers, supervisory agencies, healthcare professionals, and patients is key to maintaining the integrity of the regulatory process and enhancing patient outcomes.

## 9. Conclusions

The integration of 3D inner ear reconstruction software into otological surgery, particularly in cochlear implantation, revolutionizes surgical precision and patient outcomes, both pre- and post-operatively. By enabling detailed pre-operative visualization and simulation, this technology enhances implant placement planning, reduces intra-operative risks, and drives continuous improvement in surgical techniques. With ongoing advancements and increased accessibility, it holds promise for democratizing access to high-quality cochlear implantation services globally. Moreover, it could further augment its capabilities through artificial intelligence and machine learning, paving the way for even more precise and effective surgical outcomes in the future.

## Data Availability

No new data were created or analyzed in this study. Data sharing is not applicable to this article.
